# Supplementary material for: The ecology and epidemiology of malaria parasitism in wild chimpanzee reservoirs
Source: Commun Biol. 2022 Sep 27;5:1020. doi: 10.1038/s42003-022-03962-0 (PMC9515101; doi:10.1038/s42003-022-03962-0)
Supplement: Supplementary file 2 — Supplementary Information [file 42003_2022_3962_MOESM2_ESM.pdf]

The ecology and epidemiology of malaria parasitism in wild chimpanzee reservoirs

Scully et. al

Supplementary materials

Supplementary Table 1 | Summary of ecological and demographic parameters analyzed in the Kanyawara GLMM. Average parameter values are presented with standard deviation listed in parentheses.

ID	N	Pos	%Pos	Sex	Age	Date range	MAT[a]	ITV[b]	RF[c]
AE	16	13	81.3%	F	1.8 (0.5)	06/18/2014 - 08/06/2016	20.9 (0.6)	9.9 (1.1)	5.4 (3.8)
AJ	3	0	0.0%	M	39.6 (0.6)	06/18/2013 - 06/11/2014	20.6 (0.3)	10.1 (1.9)	4.9 (2.4)
AL	17	0	0.0%	F	32.9 (0.7)	06/24/2013 - 04/28/2016	20.9 (0.7)	10.4 (1.7)	4.5 (3.1)
AN	25	17	68.0%	F	6.0 (0.8)	06/24/2013 - 08/04/2016	21.1 (0.8)	11.2 (2.1)	3.4 (2.9)
AT	25	3	12.0%	M	15.4 (1.0)	06/08/2013 - 08/04/2016	21.0 (0.7)	11.1 (1.8)	3.1 (2.9)
AZ	23	7	30.4%	M	10.4 (0.9)	06/18/2013 - 08/04/2016	20.9 (0.7)	10.8 (1.6)	3.7 (3.3)
BB	14	1	7.1%	M	48.8 (1.2)	06/17/2013 - 07/19/2016	21.1 (0.9)	11.4 (1.3)	3.6 (3.9)
BL	18	3	16.7%	F	54.8 (0.9)	06/18/2013 - 04/08/2016	20.9 (0.8)	10.8 (1.7)	4.3 (3.0)
BO	20	10	50.0%	M	11.5 (1.0)	07/08/2013 - 08/01/2016	20.8 (0.7)	10.7 (1.7)	3.8 (3.4)
BT	20	12	60.0%	M	5.3 (0.7)	07/28/2013 - 08/15/2016	21.0 (0.8)	10.8 (1.5)	4.3 (3.6)
DL	4	0	0.0%	F	13.8 (0.3)	01/04/2016 - 08/03/2016	21.0 (0.1)	11.4 (0.2)	1.3 (0.5)
ES	25	5*	20.0%	M	20.7 (1.0)	06/05/2013 - 07/25/2016	20.9 (0.8)	11.1 (2.0)	3.6 (3.2)
GG	14	8	57.1%	F	13.1 (0.4)	02/14/2015 - 07/07/2016	20.8 (0.8)	9.9 (2.0)	5.0 (4.0)
LK	25	0	0.0%	M	32.9 (0.9)	06/08/2013 - 08/05/2016	21.0 (0.7)	11.1 (1.9)	3.4 (3.2)
LL	11	9	81.8%	F	2.8 (0.6)	07/17/2014 - 07/22/2016	20.8 (0.5)	10.6 (1.9)	4.1 (3.2)
LN	18	2	11.1%	F	17.7 (1.0)	06/08/2013 - 07/22/2016	20.8 (0.6)	10.7 (1.5)	4.3 (3.3)
ML	18	3	16.7%	F	17.6 (1.0)	08/03/2013 - 07/23/2016	20.6 (0.5)	10.6 (1.6)	4.3 (3.4)
MM	22	15	68.2%	F	3.8 (0.5)	06/11/2014 - 08/08/2016	21.2 (0.9)	11.2 (1.7)	3.6 (3.5)
MN	14	11	78.6%	M	6.7 (0.5)	07/12/2014 - 04/06/2016	21.1 (0.8)	10.6 (1.7)	4.3 (3.8)
MU	2	0	0.0%	F	43.0 (0.1)	06/06/2013 - 08/17/2013	20.6 (0.6)	11.4 (0.2)	1.8 (2.4)
MX	15	2	13.3%	M	17.5 (0.7)	08/17/2013 - 04/06/2016	20.9 (0.9)	10.8 (1.9)	3.9 (3.2)
NP	29	2*	6.9%	F	15.3 (0.9)	06/04/2013 - 08/01/2016	21.1 (0.8)	11.1 (2.0)	3.3 (2.9)
NT	12	0	0.0%	F	1.3 (0.4)	06/02/2015 - 07/27/2016	21.2 (0.9)	11.5 (1.8)	3.8 (3.6)
OB	20	12	60.0%	M	3.7 (0.4)	01/30/2015 - 08/05/2016	21.1 (0.7)	11.1 (2.0)	3.6 (3.7)
OG	21	6	28.6%	M	14.1 (1.0)	06/11/2013 - 08/03/2016	20.8 (0.7)	10.7 (1.8)	3.8 (3.1)
OL	20	9	45.0%	F	6.0 (0.9)	06/13/2013 - 03/18/2016	20.8 (0.6)	10.5 (1.7)	3.9 (3.0)
OM	20	11	55.0%	F	9.9 (0.9)	06/18/2013 - 08/04/2016	20.9 (0.8)	11.0 (2.2)	3.9 (3.4)
OP	11	8	72.7%	F	1.7 (0.3)	03/26/2015 - 03/27/2016	21.2 (0.9)	11.3 (1.4)	3.5 (2.0)
OT	22	1	4.5%	F	17.3 (0.9)	06/13/2013 - 08/04/2016	21.0 (0.7)	11.0 (2.1)	4.2 (3.9)
OU	17	1	5.9%	F	35.8 (0.9)	06/19/2013 - 04/30/2016	21.0 (0.8)	10.9 (2.1)	3.6 (2.6)
PB	18	0	0.0%	M	20.1 (1.0)	06/05/2013 - 08/05/2016	20.7 (0.5)	10.7 (1.9)	4.3 (3.5)
PL	3	1	33.3%	F	1.4 (0.4)	09/16/2015 - 07/20/2016	21.1 (0.5)	11.8 (0.1)	2.1 (2.7)
PO	25	4	16.0%	F	15.7 (1.0)	06/04/2013 - 08/11/2016	21.0 (0.7)	11.1 (1.8)	3.5 (3.2)

QK	3	1	33.3%	F	1.2 (0.5)	08/18/2015 - 07/17/2016	20.4 (0.4)	10.0 (1.5)	5.9 (6.4)
QT	15	0	0.0%	F	22.6 (1.2)	06/04/2013 - 08/09/2016	20.8 (0.6)	10.6 (1.3)	4.1 (3.4)
QV	24	11	45.8%	M	6.8 (0.9)	06/19/2013 - 08/09/2016	20.9 (0.7)	10.8 (1.8)	4.0 (3.2)
RD	18	0	0.0%	F	19.0 (0.8)	07/01/2013 - 08/02/2016	21.0 (0.8)	11.2 (1.8)	3.9 (3.4)
RS	1	0	0.0%	M	1.1 (NA)	12/05/2015 - 12/05/2015	20.1 (NA)	8.3 (NA)	8.0 (NA)
TG	22	2	9.1%	F	34.8 (1.0)	06/21/2013 - 08/11/2016	20.9 (0.7)	11.2 (1.8)	3.8 (3.2)
TJ	20	3	15.0%	M	19.6 (1.0)	06/04/2013 - 08/10/2016	20.9 (0.7)	11.0 (1.9)	3.0 (3.1)
TR	19	14	73.7%	F	4.0 (0.7)	07/31/2013 - 08/11/2016	21.1 (0.7)	10.9 (1.8)	3.8 (3.8)
TS	16	1	6.3%	F	10.1 (1.0)	06/21/2013 - 04/05/2016	20.9 (0.8)	10.8 (2.0)	3.8 (3.5)
TT	23	3	13.0%	M	14.5 (1.0)	06/21/2013 - 08/06/2016	20.9 (0.7)	10.9 (2.0)	3.5 (3.1)
TU	4	1	25.0%	M	53.0 (0.1)	06/17/2013 - 08/12/2013	20.5 (0.3)	11.7 (0.6)	2.7 (0.9)
UK	3	1	33.3%	F	2.3 (0.0)	07/29/2013 - 07/31/2013	20.2 (0.0)	11.2 (0.1)	2.7 (0.0)
UM	13	5	38.5%	F	33.6 (1.2)	07/09/2013 - 07/12/2016	20.9 (0.9)	11.0 (1.8)	4.4 (4.3)
UN	14	8	57.1%	M	10.5 (1.1)	06/08/2013 - 07/19/2016	21.0 (0.8)	11.4 (1.9)	3.5 (4.1)
WA	16	4	25.0%	F	23.7 (0.8)	06/06/2013 - 03/07/2016	21.1 (0.8)	11.5 (2.4)	3.9 (3.9)
WC	22	16	72.7%	M	6.8 (0.9)	07/12/2013 - 07/14/2016	20.8 (0.7)	10.2 (1.5)	4.3 (3.2)
WE	17	10	58.8%	F	8.2 (0.5)	06/06/2014 - 06/21/2016	21.0 (0.8)	10.7 (2.1)	4.7 (4.1)
WL	20	7	35.0%	F	22.8 (1.0)	06/08/2013 - 07/14/2016	20.8 (0.7)	10.6 (1.7)	4.0 (3.4)
WO	10	6	60.0%	F	1.1 (0.3)	11/16/2015 - 07/23/2016	21.0 (0.9)	10.9 (2.2)	3.4 (4.0)
WZ	11	3*	27.3%	M	3.3 (0.5)	06/11/2014 - 06/21/2016	20.7 (0.5)	10.4 (1.6)	6.3 (3.9)
YB	20	1	5.0%	M	41.7 (1.0)	06/27/2013 - 07/19/2016	20.9 (0.5)	10.7 (1.7)	3.9 (3.2)
Total	878	273	31.1%		16.6 (12.6)	06/04/2013 - 08/15/2016	20.9 (0.7)	10.9 (1.8)	3.9 (3.3)

[a]MAT: mean ambient temperature (average of estimates recorded 30 days prior to sample collection; measured directly)
[b]ITV: intra-day temperature variation (average of estimates recorded 30 days prior to sample collection; measured directly)
[c]RF: rainfall (average of estimates recorded 30 days prior to sample collection; measured directly)

Supplementary Table 2 | Summary of ecological parameters analyzed in the Pan-African GLMM. Average parameter values are presented with standard deviation listed in parentheses.

Site	N (int*)	Pos	%Pos	Date range	MAT[a]	ITV[b]	FC[c]	RF[d]
AM	37 (23)	0	0.0%	01/15/2005 - 08/04/2005	24.7 (0.8)	8.5 (0.2)	90	4.3 (1.4)
AN	15 (0)	0	0.0%	03/16/2007 - 03/26/2007	23.7 (0.1)	11.5 (0.2)	47	2.9 (0.3)
AZ	32 (31)	0	0.0%	09/05/2007 - 05/20/2016	26.0 (0.7)	8.0 (0.5)	70	5.5 (1.0)
BA	258 (0)	61	23.6%	12/23/2005 - 11/08/2009	23.9 (0.6)	8.7 (0.8)	99	3.9 (2.1)
BB	33 (0)	8	24.2%	04/10/2003 - 06/08/2003	24.3 (0.6)	8.3 (0.3)	90	5.2 (0.2)
BD	6 (0)	0	0.0%	01/25/2001 - 02/10/2002	21.2 (0.4)	11.5 (0.4)	81	0.3 (0.1)
BF	31 (0)	20	64.5%	12/04/2006 - 07/04/2007	24.8 (0.6)	9.1 (0.6)	90	3.3 (2.0)
BG	42 (0)	7	16.7%	05/19/2014 - 05/31/2014	23.0 (0.0)	9.5 (0.1)	100	3.5 (0.3)
BI	93 (87)	10	10.8%	03/15/2003 - 12/03/2007	24.2 (0.4)	8.6 (0.3)	90	5.9 (2.3)
BL	7 (0)	0	0.0%	03/22/2007 - 05/03/2007	23.8 (0.5)	9.4 (0.2)	100	6.1 (1.3)
BO	29 (0)	0	0.0%	01/15/2012 - 03/10/2012	21.8 (0.8)	11.7 (0.4)	54	0.3 (0.7)
BQ	67 (0)	13	19.4%	02/05/2003 - 08/08/2004	22.8 (0.3)	8.4 (0.4)	95	4.3 (2.1)
CP	17 (0)	0	0.0%	03/24/2004 - 03/28/2010	24.5 (0.9)	8.5 (0.7)	90	3.9 (1.0)
DG	44 (0)	6	13.6%	05/20/2004 - 10/14/2004	23.2 (0.4)	8.4 (0.6)	82	6.0 (0.8)
DP	157 (0)	31	19.7%	02/15/2003 - 03/27/2004	22.9 (0.4)	8.8 (0.6)	90	3.6 (2.2)
EB	25 (0)	1	4.0%	03/26/2007 - 04/17/2008	22.1 (0.3)	8.6 (0.6)	87	3.0 (2.3)
EK	18 (0)	1	5.6%	07/08/2004 - 08/18/2004	22.7 (0.1)	8.4 (0.2)	90	5.0 (1.0)
EN	26 (26)	1	3.8%	02/29/2016 - 04/29/2016	26.9 (0.3)	8.8 (0.6)	100	4.2 (1.8)
EP	10 (0)	1	10.0%	01/30/2006 - 09/05/2006	24.6 (0.6)	8.7 (0.2)	100	2.6 (1.4)
GI	42 (0)	0	0.0%	04/14/2009 - 09/15/2009	13.3 (2.0)	11.6 (1.4)	37	1.2 (0.6)
GM	169 (0)	0	0.0%	11/06/2000 - 05/24/2005	24.5 (0.9)	8.1 (0.9)	20	2.3 (2.2)
GO	2 (0)	0	0.0%	12/15/2005 - 12/29/2005	21.5 (0.7)	11.0 (0.4)	77	0.4 (0.1)
GT	168 (0)	56	33.3%	08/30/2003 - 06/22/2005	24.9 (0.5)	8.5 (0.5)	100	4.7 (1.8)
IS	3 (0)	0	0.0%	12/05/2005 - 12/15/2005	22.1 (0.3)	10.5 (0.1)	100	1.3 (0.3)
KA	104 (0)	32	30.8%	12/12/2005 - 10/03/2007	23.7 (0.8)	8.9 (0.5)	99	3.8 (1.6)
KB	46 (0)	6	13.0%	03/09/2003 - 04/19/2003	22.1 (0.1)	10.4 (0.2)	89	2.3 (0.8)
KO	45 (0)	1	2.2%	02/24/2008 - 07/25/2009	25.0 (0.8)	9.6 (1.2)	29	3.0 (1.1)
KS	11 (11)	1	9.1%	06/10/2004 - 01/18/2006	25.6 (0.6)	11.2 (1.1)	16	4.1 (1.8)
KY	34 (0)	0	0.0%	07/02/2014 - 07/08/2014	22.6 (0.1)	11.7 (0.1)	16	2.6 (0.1)
LB	13 (0)	3	23.1%	03/04/2003 - 01/10/2004	24.8 (0.5)	8.2 (0.2)	100	2.8 (0.4)
LH	3 (0)	0	0.0%	06/20/2007 - 12/07/2007	24.3 (0.8)	8.1 (0.1)	76	6.0 (3.1)
LU	120 (98)	12	10.0%	03/31/2007 - 06/13/2008	23.7 (0.6)	9.6 (1.1)	16	5.9 (1.6)
MB	16 (0)	5	31.3%	03/10/2003 - 12/15/2003	23.6 (0.7)	8.3 (0.5)	90	4.3 (1.6)
MD	4 (0)	0	0.0%	04/04/2008 - 04/09/2008	23.4 (0.0)	9.4 (0.2)	79	2.8 (0.0)
MF	13 (0)	0	0.0%	08/21/2005 - 08/25/2005	24.3 (0.0)	7.5 (0.0)	56	6.7 (0.2)
MH	26 (0)	0	0.0%	02/10/2002 - 11/25/2003	20.1 (0.3)	11.6 (0.7)	47	2.5 (3.0)
MK	4 (0)	2	50.0%	04/05/2008 - 05/08/2008	21.1 (1.3)	9.8 (0.7)	90	5.4 (1.0)
MP	1 (0)	0	0.0%	12/02/2005 - 12/02/2005	23.6 (NA)	10.1 (NA)	68	1.1 (NA)

MT	56 (0)	7	12.5%	05/25/2003 - 05/29/2004	22.2 (0.6)	10.2 (1.2)	59	2.4 (2.9)
MU	34 (0)	15	44.1%	11/27/2005 - 03/12/2007	24.2 (0.4)	9.6 (0.7)	100	5.6 (2.8)
NB	62 (0)	0	0.0%	12/14/2011 - 03/06/2012	22.8 (0.4)	9.5 (0.2)	95	0.3 (0.4)
NY	27 (0)	0	0.0%	08/14/2002 - 02/11/2004	13.5 (1.3)	11.2 (0.6)	51	2.3 (1.5)
ON	40 (0)	16	40.0%	01/14/2007 - 02/20/2007	23.2 (0.2)	9.1 (0.1)	100	1.6 (0.6)
OP	4 (0)	0	0.0%	06/20/2007 - 08/20/2007	22.9 (0.9)	9.1 (0.6)	98	4.9 (1.9)
PA	86 (80)	28	32.6%	05/27/2003 - 12/13/2010	25.0 (0.4)	8.8 (0.4)	100	5.3 (1.2)
PO	7 (0)	0	0.0%	08/28/2004 - 12/08/2005	23.6 (0.1)	8.9 (0.4)	55	5.5 (2.5)
SL	1 (0)	0	0.0%	06/28/2005 - 06/28/2005	23.8 (NA)	7.0 (NA)	49	3.2 (NA)
UB	94 (0)	4	4.3%	12/27/2005 - 07/16/2009	24.4 (0.7)	8.6 (0.5)	100	3.4 (2.0)
UG	65 (0)	0	0.0%	11/13/2009 - 03/05/2010	20.7 (0.9)	10.2 (0.8)	51	4.4 (1.9)
VM	6 (0)	2	33.3%	04/25/2008 - 04/25/2008	21.6 (0.0)	10.3 (0.0)	74	4.3 (0.0)
WA	115 (106)	26	22.6%	02/22/2003 - 04/28/2006	23.6 (0.8)	8.6 (0.7)	93	5.6 (1.7)
WB	1 (0)	0	0.0%	11/30/2007 - 11/30/2007	22.6 (NA)	10.6 (NA)	37	6.7 (NA)
WE	26 (0)	7	26.9%	05/19/2004 - 05/28/2004	23.9 (0.3)	7.6 (0.1)	20	5.4 (0.5)
WL	38 (37)	11	28.9%	02/26/2004 - 04/16/2005	23.8 (0.2)	8.9 (0.3)	100	7.0 (1.9)
YW	3 (0)	2	66.7%	01/22/2008 - 01/22/2008	24.4 (0.0)	8.5 (0.0)	85	0.9 (0.0)
Total	2436 (499)	396	16.3%	11/06/2000 - 05/20/2016	23.4 (2.1)	9.1 (1.1)	77.1 (30.0)	3.9 (2.3)

*Int: Number of replicates to which an intensive PCR protocol, in which samples were screened in 8-10 replicates, was applied.

[a]MAT: mean ambient temperature (average of estimates recorded 30 days prior to sample collection; estimated from remote-sensing MODIS LST[1] dataset)

[b]ITV: intra-day temperature variation (average of estimates recorded 30 days prior to sample collection; estimated from remote-sensing MODIS LST[1] dataset)

[c]FC: forest cover (derived from Hansen et al.[2] dataset)

[d]RF: rainfall (average of estimates recorded 30 days prior to sample collection; estimated from remote-sensing GPCP[3] dataset)

Supplementary Data 1 | Accession numbers of mtDNA reference sequences included in Figure 2.
See attached Microsoft Excel file.

Supplementary Data 2 | Accession numbers of mtDNA reference sequences included in Figure 3.
See attached Microsoft Excel file.

Supplementary Data 3 | Accession numbers of mtDNA sequences newly generated in this study.
See attached Microsoft Excel file.

Supplementary Data 4 | GenBank accession numbers of chimpanzee *Plasmodium* sequences
See attached Microsoft Excel file.

Supplementary References

1. Tatem, A. J., Goetz, S. J. & Hay, S. I. Terra and Aqua: New data for epidemiology and public health. *International Journal of Applied Earth Observation and Geoinformation* **6**, 33–46 (2004).
2. Hansen, M. C. *et al.* High-Resolution Global Maps of 21st-Century Forest Cover Change. *Science (1979)* **342**, 850–853 (2013).
3. Adler, R. F. *et al.* The Version-2 Global Precipitation Climatology Project (GPCP) Monthly Precipitation Analysis (1979–Present). *Journal of Hydrometeorology* **4**, 1147–1167 (2003).
