# Supplementary material for: The ecology and epidemiology of malaria parasitism in wild chimpanzee reservoirs
Source: Commun Biol. 2022 Sep 27;5:1020. doi: 10.1038/s42003-022-03962-0 (PMC9515101; doi:10.1038/s42003-022-03962-0)
Supplement: Supplementary file 3 — Description of Additional Supplementary Files [file 42003_2022_3962_MOESM3_ESM.pdf]

Description of Additional Supplementary Files

File name: Supplementary Data 1-4
Description:

Supplementary Data 1: Accession numbers of mtDNA reference sequences included in Figure 2.
Supplementary Data 2: Accession numbers of mtDNA reference sequences included in Figure 3.
Supplementary Data 3: Accession numbers of mtDNA sequences newly generated in this study.
Supplementary Data 4: GenBank accession numbers of chimpanzee *Plasmodium* sequences.
