# Supplementary material for: The ecology and epidemiology of malaria parasitism in wild chimpanzee reservoirs
Source: Commun Biol. 2022 Sep 27;5:1020. doi: 10.1038/s42003-022-03962-0 (PMC9515101; doi:10.1038/s42003-022-03962-0)
Supplement: Supplementary file 5 — Reporting Summary [file 42003_2022_3962_MOESM5_ESM.pdf]

nature research

Corresponding author(s): Manoj T. Duraisingh
Beatrice H. Hahn

Last updated by author(s): Jul 14, 2022

Reporting Summary

Nature Research wishes to improve the reproducibility of the work that we publish. This form provides structure for consistency and transparency in reporting. For further information on Nature Research policies, see our Editorial Policies and the Editorial Policy Checklist.

Statistics

For all statistical analyses, confirm that the following items are present in the figure legend, table legend, main text, or Methods section.

n/a	Confirmed	
☐	☒	The exact sample size (*n*) for each experimental group/condition, given as a discrete number and unit of measurement
☐	☒	A statement on whether measurements were taken from distinct samples or whether the same sample was measured repeatedly
☐	☒	The statistical test(s) used AND whether they are one- or two-sided<br>*Only common tests should be described solely by name; describe more complex techniques in the Methods section.*
☐	☒	A description of all covariates tested
☒	☐	A description of any assumptions or corrections, such as tests of normality and adjustment for multiple comparisons
☐	☒	A full description of the statistical parameters including central tendency (e.g. means) or other basic estimates (e.g. regression coefficient) AND variation (e.g. standard deviation) or associated estimates of uncertainty (e.g. confidence intervals)
☐	☒	For null hypothesis testing, the test statistic (e.g. *F*, *t*, *r*) with confidence intervals, effect sizes, degrees of freedom and *P* value noted<br>*Give P values as exact values whenever suitable.*
☐	☒	For Bayesian analysis, information on the choice of priors and Markov chain Monte Carlo settings
☒	☐	For hierarchical and complex designs, identification of the appropriate level for tests and full reporting of outcomes
☒	☐	Estimates of effect sizes (e.g. Cohen's *d*, Pearson's *r*), indicating how they were calculated

Our web collection on statistics for biologists contains articles on many of the points above.

Software and code

Policy information about availability of computer code

Data collection	No commercial or proprietary software was used for data collection in this study.
Data analysis	For analysis of malaria parasitism within the Kanyawara chimpanzee community, we trimmed sequences to 863 bps and used Geneious aligner (version 10) to align sequences to a set of phylogenetically informative reference sequences. We used jModelTest (version 2.0) to identify the best-fit nucleotide substitution model and MrBayes (version 3.2.6) to generate Bayesian posterior probabilities. All statistical analyses were performed using open-source packages in R (version 3.4.3) as referenced in the methods section. The scripts used to implement these analyses are available from the corresponding authors upon request.

For manuscripts utilizing custom algorithms or software that are central to the research but not yet described in published literature, software must be made available to editors and reviewers. We strongly encourage code deposition in a community repository (e.g. GitHub). See the Nature Research guidelines for submitting code & software for further information.

Data

Policy information about availability of data

All manuscripts must include a data availability statement. This statement should provide the following information, where applicable:

- Accession codes, unique identifiers, or web links for publicly available datasets
- A list of figures that have associated raw data
- A description of any restrictions on data availability

Genetic sequence data generated in this study has been deposited at NCBI GenBank under the accession numbers MW228501-MW228790, OL691962-OL691978, (see Table S2 for sequence details). Previously published sequences analyzed in this study are included in Tables S4-S6. All other datasets generated in this study are available from the corresponding authors upon request.

Field-specific reporting

Please select the one below that is the best fit for your research. If you are not sure, read the appropriate sections before making your selection.

☐ Life sciences ☐ Behavioural & social sciences ☒ Ecological, evolutionary & environmental sciences

For a reference copy of the document with all sections, see nature.com/documents/nr-reporting-summary-flat.pdf

Ecological, evolutionary & environmental sciences study design

All studies must disclose on these points even when the disclosure is negative.

Study description

In this study, we used a combination of longitudinal and cross-sectional sampling strategies to collect 3,314 fecal samples from wild chimpanzees for molecular analyses of malaria parasitism.

Research sample

In this study, we used a combination of longitudinal and cross-sectional sampling strategies to collect 3,314 fecal samples from wild chimpanzees for molecular analyses of malaria parasitism.

For longitudinal analyses, 878 fecal samples were collected from 54 chimpanzees inhabiting the Kanyawara chimpanzee community, located in Kibale National Park, western Uganda. This cohort of wild chimpanzees was habituated to human observation by RWW and has been under continuous direct observation since 1987. All Kanyawara study subjects are identifiable by both morphological appearance and microsatellite genotype. At this field site, researchers and field assistants conduct focal follows of individual chimpanzees on a daily basis and record individual-level behavior, party-level behavior, social affiliation data, and biological samples, among other parameters. Demographic information (i.e., age and sex) of all chimpanzees sampled in this cohort is outlined in Figure S1.

For cross-sectional analyses, 2,436 chimpanzee fecal samples were collected from 55 chimpanzee field sites, distributed across equatorial Africa (see Fig. 1; Fig. S2). This dataset included 1,936 wild chimpanzee samples, previously collected for molecular studies of simian retroviruses, and 500 samples that were newly collected for these analyses (see Table 1).

Sampling strategy

Samples were collected opportunistically by researchers and field assistants either: (A) conducting focal follows of individual wild chimpanzees habituated to human observation as part of long-term field studies, or (B) collected from non-habituated chimpanzees by field assistants during ape and biodiversity surveys.

Data collection

Longitudinal study (Kanyawara chimpanzee community; western Uganda): Fecal samples were opportunistically collected only upon direct observation of defecation and only when a positive identification was certain—were preserved in RNAlater (1:1 vol/vol) and stored at -20°C until exportation and DNA extraction.

Cross-sectional analysis (55 sites across equatorial Africa): This dataset comprised a combination of samples that were either opportunistically collected from habituated chimpanzees occupying long-term research sites or opportunistically collected from non-habituated chimpanzees during ape and biodiversity surveys.

Timing and spatial scale

Longitudinal study: 1 site (i.e., Kanyawara chimpanzee community) in Western Uganda; 06/04/2013 - 08/15/2016

Cross-sectional study: 55 sites across equatorial Africa (see Fig. 1; Fig. S2); 11/16/2000 - 05/20/2016

Data exclusions

Samples were only included in this dataset if collection dates were recorded and corresponding ecological variables (see below) were available.

Reproducibility

In this study, we used a combination of longitudinal and cross-sectional sampling strategies to collect fecal samples from wild chimpanzees for molecular analyses of malaria parasitism. Due to the observational nature and large scale of this study, sample collection cannot be replicated per se. However, cross-sectional and longitudinal samplings strategies, which focused upon different chimpanzee populations, produced internall consistent results.

Randomization

In this study, we used a combination of longitudinal and cross-sectional sampling strategies to collect fecal samples from wild chimpanzees for molecular analyses of malaria parasitism. Due to the observational nature of this study, randomization was not possible.

Blinding

Chimpanzee fecal samples were collected opportunistically upon observation of defecation. Samples were given an alphanumeric identifier during sequencing and

Did the study involve field work? ☒ Yes ☐ No

Field work, collection and transport

Field conditions

Mean ambient temperature, ambient temperature variation, and rainfall values associated with each sample are summarized in Figures S1 and S2.

Location

Longitudinal study: 1 site (i.e., Kanyawara chimpanzee community) in Western Uganda; 06/04/2013 - 08/15/2016
Cross-sectional study: 55 sites across equatorial Africa (see Fig. 1; Fig. S2); 11/16/2000 - 05/20/2016.

Access & import/export	Collection and exportation of chimpanzee fecal samples used in this study was accomplished with relevant research permissions and exportation approvals provided by the Uganda Wildlife Authority and the Uganda National Council for Science and Technology.
Disturbance	All sample collection was non-invasive and opportunistic, resulting in minimal disturbance to the study population.

Reporting for specific materials, systems and methods

We require information from authors about some types of materials, experimental systems and methods used in many studies. Here, indicate whether each material, system or method listed is relevant to your study. If you are not sure if a list item applies to your research, read the appropriate section before selecting a response.

Materials & experimental systems

n/a	Involved in the study
☒	☐ Antibodies
☒	☐ Eukaryotic cell lines
☒	☐ Palaeontology and archaeology
☐	☒ Animals and other organisms
☒	☐ Human research participants
☒	☐ Clinical data
☒	☐ Dual use research of concern

Methods

n/a	Involved in the study
☒	☐ ChIP-seq
☒	☐ Flow cytometry
☒	☐ MRI-based neuroimaging

Animals and other organisms

Policy information about studies involving animals; ARRIVE guidelines recommended for reporting animal research

Laboratory animals	This study did not involve laboratory animals.
Wild animals	In this study, we used a combination of longitudinal and cross-sectional sampling strategies to collect 3,314 fecal samples from wild chimpanzees for molecular analyses of malaria parasitism.<br><br>For longitudinal analyses, 878 fecal samples were collected from 54 chimpanzees inhabiting the Kanyawara chimpanzee community, located in Kibale National Park, western Uganda. This cohort of wild chimpanzees was habituated to human observation by RWW and has been under continuous direct observation since 1987. All Kanyawara study subjects are identifiable by both morphological appearance and microsatellite genotype. At this field site, researchers and field assistants conduct focal follows of individual chimpanzees on a daily basis and record individual-level behavior, party-level behavior, social affiliation data, and biological samples, among other parameters. Demographic information (i.e., age and sex) of all chimpanzees sampled in this cohort is outlined in Figure S1.<br><br>For cross-sectional analyses, 2,436 chimpanzee fecal samples were collected from 55 chimpanzee field sites, distributed across equatorial Africa (see Fig. 1; Fig. S2). This dataset included 1,936 wild chimpanzee samples, previously collected for molecular studies of simian retroviruses, and 500 samples that were newly collected for these analyses (see Table 1).
Field-collected samples	After collection, chimpanzee fecal samples were preserved in RNAlater (1:1 vol/vol) and stored at -20°C until exportation and DNA extraction.
Ethics oversight	No ethical approval or guidance was required for this study, because all sample collection was non-invasive and opportunistic, resulting in minimal disturbance to the study population.

Note that full information on the approval of the study protocol must also be provided in the manuscript.
